# Cortisol Imbalance and Fear Learning in PTSD: Therapeutic Approaches to Control Abnormal Fear Responses

**DOI:** 10.2174/1570159X23666250123142526

**Published:** 2025-01-23

**Authors:** Simone Battaglia, Chiara Di Fazio, Sara Borgomaneri, Alessio Avenanti

**Affiliations:** 1 Centro studi e ricerche in Neuroscienze Cognitive, Dipartimento di Psicologia “Renzo Canestrari”, Alma Mater Studiorum Università di Bologna, Cesena Campus, Cesena, Italy;; 2 Dipartimento di Psicologia, Università degli Studi di Torino, Torino, Italy;; 3 Neuropsicology and Cognitive Neuroscience Research Center (CINPSI Neurocog), Universidad Católica del Maule, Talca, Chile

**Keywords:** Post-traumatic stress disorder (PTSD), hypothalamic-pituitary-adrenal (HPA) axis, cortisol dysregulation, dexamethasone, fear learning, fear extinction

## Abstract

Post-Traumatic Stress Disorder (PTSD) is mainly characterized by dysregulated fear responses, including hyperarousal and intrusive re-experiencing of traumatic memories. This work delves into the intricate interplay between abnormal fear responses, cortisol dysregulation, and the Hypothalamic-Pituitary-Adrenal (HPA) axis, elucidating their role in the manifestation of PTSD. Given the persistent nature of PTSD symptoms and the limitations of conventional therapies, innovative interventions are urgently needed. One promising avenue of research revolves around the modulation of cortisol through targeting receptors, with dexamethasone emerging as a critical agent capable of reducing cortisol levels, thus potentially aiding in the extinction of fear. In this study, we emphasize the need for innovative interventions in the neuropharmacological treatment of PTSD, focusing on cortisol modulation and its impact on fear regulation mechanisms. The complex interplay between the HPA axis, cortisol modulation, and fear dysregulation not only broadens our comprehension but also reveals promising paths to enhance therapeutic outcomes for individuals struggling with PTSD, underscoring a crucial need for more effective treatment strategies.

## 1 INTRODUCTION

Post-Traumatic Stress Disorder (PTSD) is a complex and debilitating psychiatric condition characterized by dysregulated fear responses, which may manifest in individuals who have experienced or witnessed a traumatic event [1]. Clinical features include intrusive symptoms such as flashbacks and nightmares, avoidance of trauma-related stimuli, negative alterations in mood and cognition, and heightened arousal and reactivity, all of which can significantly impact the individual’s daily functioning and quality of life [2, 3]. Fear learning and fear extinction are two complex phenomena that play a major role in PTSD [4]. Fear learning is the process by which an individual learns to associate a neutral stimulus with a traumatic event, leading to the development of a fear ‘conditioned’ response [5-7]. Fear extinction, on the other hand, is the learning process that occurs when the conditioned stimulus no longer predicts the aversive event, leading to a suppression of the fear response through newly learned associations with ‘safe’ outcomes [5]. Studying these phenomena in a controlled experimental setting is advantageous as it allows for precise assessment of behavioral and physiological responses to fear-related conditioned stimuli. Typically, human studies involve exposing participants to one or more neutral stimuli, at least one of which is then paired with aversive outcomes, such as electric shocks, to generate conditioned responses [8, 9]. These responses can subsequently be reduced to baseline levels through extinction procedures, where the stimuli are presented without adverse consequences [8].

Fear learning is evolutionarily advantageous, allowing individuals to recognize and avoid potential threats. However, when fear responses become overgeneralized, they may contribute to the development of psychiatric conditions such as PTSD, where individuals may experience persistent hyperarousal and re-experiencing of the traumatic event [3, 10-12]. In PTSD, dysregulation often stems from a failure of fear extinction, where the traumatic memory remains active, and the associated fear response is not adequately suppressed, even when it is no longer relevant [13]. In this context, reconsolidation is a key process where existing memories are initially recalled and then (re)consolidated with modifications [14]. Abnormalities in this process can lead to reinforced trauma-related memories, exacerbating fear responses and compounding the symptoms of PTSD [4, 13, 15-17].

## 2 GLUCOCORTICOID RECEPTORS AND FEAR REGULATION

Among factors such as genetics, personality traits, and the nature of traumatic events, the dysregulated fear responses in PTSD can also be associated with altered activity of Glucocorticoid Receptors (GRs) [18] and Mineralocorticoid Receptors (MRs) [19]. GRs are widely distributed throughout the brain, particularly in key regions involved in fear learning and extinction, such as the hippocampus, amygdala, and prefrontal cortex, where they play a crucial role in modulating gene transcription upon glucocorticoid activation, impacting memory consolidation, and leading to various physiological and behavioral effects [20]. In contrast, MRs, which have a higher affinity for glucocorticoids than GRs, are primarily concentrated in the hippocampus. These receptors are also involved in the modulation of stress responses and fear regulation, impacting both the threshold for stress response activation and the recovery process [21, 22]. Maintaining a balance between GR and MR activation is essential for appropriate stress responses and emotional regulation, influencing the pathophysiology of PTSD and its potential treatment strategies.

**Fig. (1):**
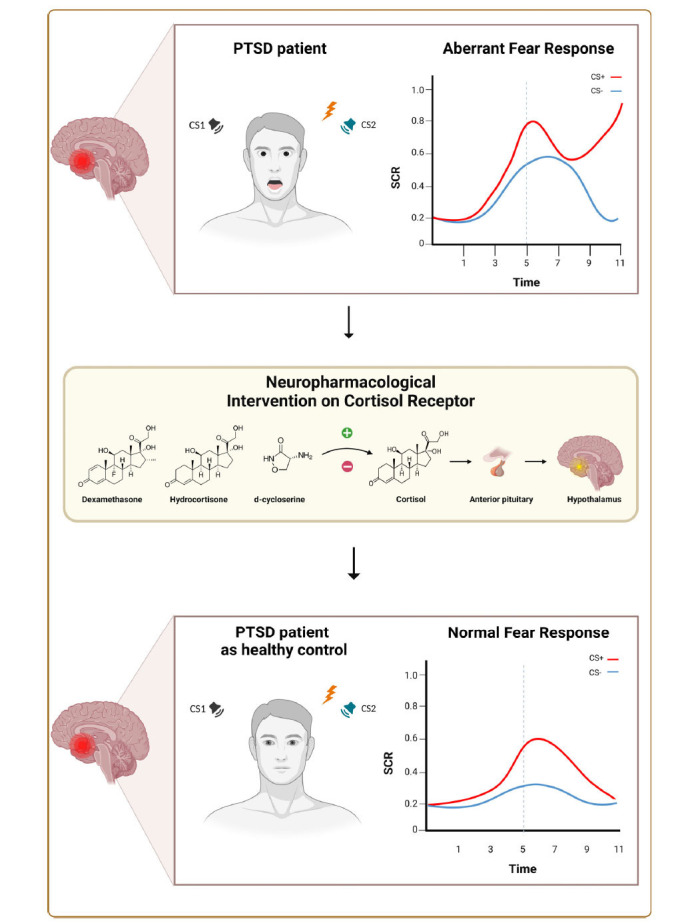
Schematic representation of the neuropharmacological mechanisms of receptors in PTSD patients, highlighting the interplay between aberrant fear responses and cortisol level dysregulation. The figure illustrates the roles of dexamethasone and hydrocortisone in modulating glucocorticoid receptor sensitivity and cortisol levels. In contrast, d-cycloserine does not directly affect cortisol levels; instead, it facilitates synaptic plasticity by acting on glutamatergic pathways, which are crucial for fear memory consolidation. Dexamethasone appears to have a more pronounced effect on fear extinction in PTSD, particularly in reversing extinction deficits, compared to hydrocortisone and d-cycloserine. These treatments aim to normalize aberrant fear responses and associated neurophysiological symptoms in PTSD. The figure was created using BioRender.com.

In the context of PTSD, dysregulation of glucocorticoid signaling has been implicated in the impaired fear extinction observed in affected individuals. Reduced activity of cortisol, the primary endogenous glucocorticoid, has been associated with heightened fear responses and difficulties in fear memory consolidation and extinction [23]. This dysregulation is closely linked to alterations in the Hypothalamic-Pituitary-Adrenal (HPA) axis, a key regulator of the stress response. Specifically, cortisol is known to have anti-inflammatory effects under normal physiological conditions. During acute stress, the activation of the HPA axis leads to increased cortisol production, which, among other functions, serves to modulate the immune response by inhibiting excessive inflammation [24-27]. This mechanism allows the body to mobilize energy and resources to cope with stressors effectively [28, 29]. However, the relationship between cortisol and inflammation is not straightforward and varies depending on the context and duration of stress exposure. In individuals with PTSD, several studies have reported dysregulation of the HPA axis, leading to altered cortisol levels [23, 30]. This dysregulation is often characterized by lower basal levels of cortisol, which is noteworthy as it may intensify inflammation. The mechanism involves the conversion of inactive cortisone to active cortisol by the enzyme 11B-HSD1 and the reverse process by 11B-HSD2 [31]. Changes in the activity of these enzymes in inflammatory conditions can lead to altered cortisol activity, contributing to increased inflammation. PTSD has been associated with a chronic low-grade inflammatory state, evidenced by increased levels of pro-inflammatory cytokines and markers such as IFN-γ, TNF-α, and C-reactive protein [32]. This state of heightened inflammation can contribute to the pathophysiology of PTSD, affecting fear memory consolidation and extinction. Importantly, growing evidence suggests that dysregulation of the inflammatory response contributes to the impaired fear extinction observed in patients with PTSD [23, 28, 33]. Therefore, the dysregulation of cortisol and inflammation in PTSD intensifies the dysregulated fear responses that are characteristic of the disorder (Fig. **1**).

Notably, the relationship between reduced cortisol activity and increased inflammation in PTSD is a topic of ongoing research, highlighting a potential link between cortisol dysregulation and inflammatory processes in the disorder. The multifaceted nature of PTSD symptomatology involves not only cortisol dysregulation and inflammation but also includes impaired fear extinction and heightened amygdala response as central components [34]. Advanced neuroimaging techniques, such as functional Magnetic Resonance Imaging (fMRI) and Magnetoencephalography (MEG), have revealed that the amygdala, a key brain region involved in processing emotions such as fear, often shows heightened activity in individuals with PTSD [35, 36]. It is thought that increased amygdala activity contributes to the disorder's symptomatology. These imaging studies not only elucidated the patterns of amygdala activation but also explored its connectivity with other critical brain regions, providing valuable insights into the neural mechanisms underlying PTSD [37]. Given the chronic nature of PTSD symptoms, there is an urgent need to explore innovative interventions beyond traditional approaches, such as cognitive-behavioral therapy and pharmacological medications [38]. The dysregulated fear responses characteristic of PTSD, including hyperarousal and re-experiencing symptoms, are a primary focus of neuropharmacological research.

## 3 NEUROPHARMACOLOGICAL INTERVENTIONS

### 3.1 Dexamethasone (DEX)

Investigations into cortisol dysregulation, particularly its increased suppression following dexamethasone administration, provide valuable insights into potential therapeutic pathways [39-41]. Dexamethasone (DEX) is a synthetic glucocorticoid that functions as an agonist at GRs, exerting significant anti-inflammatory and immunosuppressive effects [42, 43]. When administered, DEX binds to GRs and influences gene expression, suppressing endogenous cortisol production by the adrenal glands, thus reducing circulating cortisol levels in the body.

In the context of PTSD treatment, the administration of DEX is strategically used to target specific pathways involved in fear extinction to ultimately alleviate symptoms. Through its pharmacological action, DEX lowers cortisol levels, thereby modulating the stress response system. DEX’s modulation of the stress response system may facilitate symptom improvement in PTSD patients by influencing the expression of the FKBP5 gene in the brain [44, 45]. By increasing FKBP5 expression, DEX enhances the negative feedback regulation of the HPA axis, thereby reducing cortisol levels and stress reactivity [44]. This effect is dose-dependent, with low doses primarily affecting peripheral tissues, while high doses potentially cross the blood-brain barrier to exert central effects. Notably, PTSD patients often exhibit enhanced negative feedback sensitivity of the HPA axis. Consequently, low doses of DEX can induce a more pronounced suppression of cortisol levels compared to healthy individuals. This altered feedback response suggests heightened HPA axis sensitivity in PTSD, which may underlie the observed effects of DEX to exert beneficial effects. Additionally, DEX can induce changes in DNA methylation of the FKBP5 gene, which may have long-lasting effects on gene expression and stress response [46]. This action could prevent the formation of persistent fearful memories-a core feature of PTSD-and potentially enhance the process of fear extinction, thereby contributing to the alleviation of symptoms [47]. While individuals with PTSD may already have dysregulated cortisol levels, the targeted use of dexamethasone to further suppress cortisol levels serves a therapeutic purpose in enhancing fear extinction and mitigating symptoms. Furthermore, the effectiveness of DEX and other glucocorticoids in treating PTSD may also vary based on gender and age. Research indicates that sex-related differences in the HPA axis regulation and immune system function can influence the response to glucocorticoid treatments. For instance, healthy women exhibit higher GR sensitivity following *in vivo* glucocorticoid stimulation compared to men, a difference that is absent in depressed patients [48]. Additionally, exogenous cortisol has been shown to enhance aggressive behavior in females but not in males, suggesting that gender-specific neurobiological responses to cortisol could impact treatment outcomes [49]. This approach highlights the interplay between exogenous glucocorticoid administration and endogenous cortisol regulation in the context of stress-related disorders like PTSD. Exogenous glucocorticoids, such as DEX, can modulate the HPA axis by providing negative feedback to reduce endogenous cortisol production, and this suppression can help in normalizing the exaggerated stress responses seen in PTSD patients. Additionally, exogenous glucocorticoids can facilitate fear extinction processes, thereby reducing the emotional impact of traumatic memories and improving overall symptomatology [18, 50]. Similarly, in anxiety and depression, exogenous glucocorticoids can influence the HPA axis by altering cortisol levels, which may help in managing symptoms by restoring the balance of glucocorticoid signaling and reducing HPA axis hyperactivity [51, 52].

### 3.2 Hydrocortisone (HC) and D-Cycloserine (DCS)

Besides DEX, recent studies have explored other potential pharmacological interventions to enhance fear extinction learning in individuals with PTSD, focusing on the effects of cortisol (hydrocortisone when used as a pharmacological intervention, HC) and D-Cycloserine (DCS). Cortisol, a naturally occurring glucocorticoid, has been shown to facilitate fear extinction retention when administered in the form of HC after extinction training. This notion is supported by studies showing that HC administration can modulate processes of fear extinction learning and inhibit fear memory retrieval [53], likely by enhancing synaptic plasticity and memory processes. HC may reduce neuroinflammation, which is often elevated in PTSD, thereby improving fear extinction processes [54, 55]. DCS, an antibiotic with cognitive-enhancing properties due to its action as a partial agonist at the N-methyl-D-aspartate (NMDA) receptor, has also been investigated for its potential to augment fear extinction [53]. DCS enhances the activity of NMDA receptors, facilitating synaptic plasticity and the extinction of conditioned fear responses. DCS administration before or after extinction training has been shown to enhance fear extinction in animal and human studies by promoting the consolidation of extinction memories and influencing the expression of genes involved in learning and memory, as well as those associated with anxiety and stress-related disorders [56]. While DCS has shown promise in enhancing fear extinction and reducing fear reinstatement in rodent studies, its administration in humans, particularly those with PTSD, has yielded mixed results [57]. In particular, some studies suggest that administering DCS shortly before extinction learning may reduce self-reported arousal and decrease amygdala activation, thus calling into question whether it can completely erase fear memory [53, 58, 59].

## 4 NEUROPHARMACOLOGICAL TREATMENTS

The gap in the literature that this article aims to address is the need for a comprehensive understanding of how cortisol dysregulation impacts fear learning in individuals with PTSD. The review seeks to synthesize and discuss the existing evidence to provide insights into the mechanisms underlying fear regulation and the potential role of synthetic glucocorticoids in enhancing fear extinction within the context of trauma exposure and fear-related disorders. By doing so, the review aims to shed light on the complex interplay between cortisol regulation, fear responses, and PTSD symptoms, thereby identifying potential therapeutic pathways.

Within the complex literature of PTSD research and cortisol dysregulation, the study of Jovanovic *et al*. [60] emerges as a pivotal investigation, shedding light on the influence of DEX treatment on the heightened Fear-Potentiated Startle responses (FPS) - a reflexive, physiological reaction to a threat-related stimulus, used as a marker of the fear reaction - shown by individuals with PTSD (Table **1**).

The primary focus was to understand how DEX impacts the conditioned fear response, considering its regulatory role in the HPA axis. The study involved a total of 100 participants, with 54 individuals undergoing fear learning at baseline, prior to DEX treatment (No-treatment group; 16 PTSD, 38 controls), and 46 participants undergoing fear learning 10h after DEX administration (Treatment group; 17 PTSD, 29 controls). During the fear learning phase, Conditioned Stimuli (CS+) was paired with an aversive Unconditioned Stimulus (US), while other stimuli (CS-) were left unreinforced. After DEX treatment, cortisol suppression was observed across both PTSD and non-PTSD participants, with no significant differences between them in terms of cortisol levels. Furthermore, the FPS responses to both CS+ and CS- were significantly higher in PTSD individuals compared to controls. Interestingly, the pronounced fear response was markedly diminished in the treatment group, suggesting that DEX administration contributed to the reduction of fear responses in PTSD individuals. Furthermore, a negative correlation was observed between cortisol levels and FPS responses, particularly within the Treatment PTSD group, indicating that the transient suppression of the HPA axis function *via* DEX mitigated exaggerated fear in patients with PTSD. This reduction of fear responses under suppressed cortisol conditions suggests that glucocorticoids, such as cortisol, may play a crucial role in the pathological fear observed in individuals with PTSD. The observed reduction in fear responses following DEX administration presents promising clinical implications for the management of PTSD. By targeting cortisol dysregulation, DEX may offer a novel avenue to modulate the fear experienced by individuals with PTSD. Importantly, participants’ awareness of the CS+/CS- contingencies remained unaffected by DEX. The fact that cognitive aspects such as contingency awareness, which allows subjects to predict the US and associate it with the CS+, leading to fear-potentiated startle specifically to the reinforced stimulus, remain intact also has significant clinical implications. This suggests that DEX treatment may selectively target the emotional components of PTSD without interfering with cognitive performance.

To further understand the connection between cortisol regulation, fear responses, and PTSD symptoms, Michopoulos *et al*. [61], in a subsequent study, focused on investigating the influence of DEX on fear learning and extinction in individuals both with and without PTSD (Table **1**). Employing a double-blind, crossover design, the study comprised two groups: individuals with PTSD and control individuals. Within-subjects treatment conditions included placebo and DEX. FPS tests were then conducted, focusing on the eyeblink component of the acoustic startle response. During fear acquisition, participants experienced reinforced CS+, non-reinforced CS-, and broadband noise probes alone in the US. Fear extinction followed, where both CS+ and CS- were presented without the US. The results showed that DEX facilitates fear extinction and safety discrimination in PTSD in a similar manner as in controls; moreover, after DEX administration, both the PTSD and control groups showed a significant reduction in FPS to the CS+, compared to the CS-. However, during the placebo condition, the PTSD group displayed a smaller difference in FPS between CS+ and CS- compared to controls. After the DEX administration, this difference between groups disappeared. Therefore, DEX appears to have “normalized” the fear response in PTSD participants, making them behave more similarly to controls. Importantly, in the placebo condition, the PTSD group exhibited deficits in fear extinction and safety discrimination, as indicated by the lack of decrease in FPS across extinction blocks. However, these deficits were markedly reversed with DEX treatment, leading to decreased FPS across extinction blocks and improved safety discrimination, suggesting that this can help in reversing the exaggerated fear responses and deficits in fear extinction and safety discrimination observed during the placebo condition. The study also discussed potential mechanisms underlying the observed effects, such as the role of GRs and FKBP5 gene regulation in fear extinction, as well as the modulation of immune system activity by dexamethasone, addressing that dexamethasone-mediated stabilization of HPA hyperactivity and suppression of immune activation may contribute to decreased fear and enhanced extinction effects in individuals with PTSD. Interestingly, the studies of Jovanovic *et al*. [60] and Michopoulos *et al*. [61] offer distinct perspectives on the effects of DEX treatment on fear responses in individuals with PTSD, focusing on cortisol suppression and reduced fear responses post-treatment, exploring the impact of DEX on fear extinction processes, and showcasing different facets of fear regulation and PTSD symptomatology. Both studies investigated the effects of DEX treatment on fear responses in individuals with PTSD by reversing deficits in fear extinction and safety discrimination observed during the placebo condition, suggesting promising clinical implications for managing pathological fear associated with PTSD through cortisol dysregulation modulation [60, 61].

In this context, given the relation between cortisol regulation, fear responses, and PTSD symptoms, the research by Inslicht *et al*. [53] focused on the impacts of HC and DCS on individuals with PTSD (Table **1**). Participants were randomly assigned to receive one of three drug conditions: HC group, DCS group, or placebo. The experimental design comprised three distinct sessions, each targeting specific facets of the fear learning and extinction learning processes. During session 1, participants underwent habituation and fear learning. They were exposed to visual stimuli used as CS+, which were paired with US, while CS- was presented without US. In session 2, participants were administered drug doses (HC, DCS, or placebo) before exposure to CS+ and CS- devoid of aversive shocks. Finally, in session 3, the retention of extinction learning was evaluated one week after its occurrence. To assess psychophysiological responses, Skin Conductance Responses (SCRs) were measured. Results showed a consistent reduction in the SCR to both CS+ and CS-, implying successful habituation. Fear learning results revealed a pronounced acquisition of fear responding, as evidenced by a significant differential SCR response to CS+ relative to CS-. During extinction learning, differences in SCR responses to CS+ and CS- between groups were observed. The drug groups, especially DCS and HC, exhibited lower SCR variations than the placebo group. These findings showed that a single dose of HC or DCS enhanced fear extinction learning in PTSD individuals. This study held significant implications in the neuropharmacological literature of PTSD because it was the first to investigate the application of DCS in a laboratory fear conditioning paradigm within a PTSD population. Importantly, the findings suggest that DCS may have potential benefits for those with greater impairments in fear extinction. These results also hint at the possibility of using adjunctive drug treatments like HC or DCS alongside exposure therapy to facilitate the critical process of extinction learning, which is essential for effective PTSD treatment [62-67]. While promising, the clinical application of these drugs needs further exploration, as previous studies have revealedmixed results in PTSD patients [68-76].

Factors such as GR sensitivity or polymorphisms in the FKBP5 gene may play a role in determining who might benefit most from these adjunctive treatments [70, 71]. The FKBP5 gene, which encodes a protein involved in regulating the stress response, has been the subject of extensive research in the context of PTSD. Studies have shown that genetic variations in FKBP5 may interact with childhood trauma to increase the risk of developing PTSD symptoms [46, 47, 68]. Additionally, FKBP5 polymorphisms, particularly in interaction with childhood abuse, have been found to predict the severity of lifetime PTSD symptoms, particularly hyperarousal symptoms [47, 68]. Furthermore, the FKBP5 gene has been associated with brain structural alterations in response to childhood trauma, suggesting its role in stress-induced HPA-axis dysregulation [46]. The gene is also regulated *via* complex interactions among environmental stressors, genetic variants, and epigenetic modifications, contributing to diverse phenotypes related to stress-related disorders such as PTSD [44, 77-81]. Indeed, research has shown that FKBP5 gene polymorphisms, such as the rs1360780 T allele, are associated with altered stress response and increased risk for developing PTSD following trauma exposure. In this context, DEX has been found to enhance fear extinction and regulate FKBP5 expression, normalizing the stress response and improving PTSD symptoms [61]. Similarly, DCS facilitates fear extinction learning, which is crucial for effective PTSD treatment, although its direct effects on FKBP5 require further investigation [63].

## 5 DISCUSSION OF THE MAJOR FINDINGS

Given this preliminary but crucial evidence, the exploration of the neurobiological mechanisms of PTSD has highlighted the intricate interplay between aberrant fear responses and cortisol dysregulation (Fig. **1**). Discussions around the major findings from these studies emphasize the role of the HPA axis, cortisol dysregulation, pronounced hyperarousal responses, and the impairment of fear learning mechanisms as pivotal in shaping the neurophysiological symptoms of this disorder (Table **1**) [82]. PTSD presents challenges for the healthcare system due to its enduring nature and the limitations of established treatment modalities such as cognitive-behavioral therapy and pharmacotherapy. Glucocorticoids like DEX and HC and the antibiotic DCS show promise in enhancing fear extinction and modulating the stress response in PTSD treatment [53, 61, 83]. However, it is important to consider that glucocorticoids may cause significant side effects, including adrenal suppression, hyperglycemia, osteoporosis, and psychiatric complications [84]. Their potential therapeutic applications highlight the need for research to refine these treatments and develop strategies to optimize their efficacy and safety.

Investigating the interaction between the HPA axis, cortisol modulation, and fear learning dysregulation not only expands scientific knowledge but also addresses promising possibilities for enhancing therapeutic results in individuals struggling with psychiatric conditions. In this context, for example, by focusing on the neurobiological and psychological processes that contribute to dysregulated learned fear, it could be possible to develop targeted strategies to mitigate the onset of mental illness, ameliorate the symptoms, and improve outcomes for trauma survivors. Indeed, one crucial aspect may focus on preventive therapy, where a deeper comprehension of the mechanisms behind dysregulated fear responses could lead to better interventions, especially for individuals with PTSD. Recent research has explored the modulation of cortisol receptors, with dexamethasone emerging as a promising agent [39-41]. Researchers have shown particular interest in dexamethasone’s capacity to target and reduce cortisol levels, especially regarding its potentials to increase the extinction of fear [46, 47]. In light of this, steroids have also shown varying effects on fear learning, depending on cortisol levels [85], in which higher levels enhance fear memory consolidation while lower levels promote fear extinction. Although steroids are being investigated for their ability to modulate fear responses in PTSD, clinical results remain mixed, highlighting the need for further research [85-87].

In addition to classical pharmacological approaches, neurostimulation techniques are also gaining attention for their potential in treating such psychiatric conditions characterized by aberrant fear learning [88-90]. In this context, Transcranial Magnetic Stimulation (TMS) offers a promising avenue for modulating fear extinction processes in PTSD. This non-invasive technique employs magnetic fields to alter cortical excitability, enhancing or inhibiting brain activity depending on stimulation parameters. High-frequency TMS induces Long-Term Potentiation (LTP), while low-frequency TMS promotes Long-Term Depression (LTD), both of which can modify neural plasticity and connectivity [91-98].

Recent studies reveal TMS’s potential to enhance the expression of brain-derived neurotrophic factors and other genes crucial for synaptic plasticity, thereby facilitating fear extinction and forming non-fearful associations [99-105]. Furthermore, TMS modulates the prefrontal cortex's activity and its connectivity with the amygdala, a region central to fear processing. This modulation reduces hyperarousal and improves emotional regulation in PTSD patients [15,16, 106-110].

These findings underscore TMS's dual impact at both behavioral and molecular levels, offering a novel adjunctive treatment for PTSD. By addressing the neurobiological underpinnings of fear extinction, TMS holds potential to reshape clinical approaches and improve outcomes for trauma survivors [111-113].

## CONCLUSION

In conclusion, by integrating neuropharmacological and neurostimulation approaches, researchers are advancing the framework for novel treatments for PTSD, with a focus on unveiling the mechanisms behind aberrant fear extinction, cortisol modulation, and related neurobiological pathways dynamics [73, 114-120].

## Figures and Tables

**Table 1:** Summary of findings in studies with the administration of dexamethasone, hydrocortisone, and d-cycloserine in the study of neuropharmacological mechanisms of PTSD.

**Study**	**Group (N)**	**Pharmacological Treatment**	**Mechanism of Action**	**Phase of Fear Learning**	**CS/US**	**Psychophysiological Measure**	**Main Findings**
Jovanovic *et al*., [60]	No-treatment - placebo (16 PTSD, 38 controls) Treatment - DEX (17 PTSD, 29 controls)	0.5 mg dose of DEX, Placebo	DEX is an agonist of glucocorticoid receptors	Habituation, Acquisition	Geometric figures/ Air blast	EMG	PTSD individuals showed greater fear potentiation to the CS+ compared to controls. In the treatment group, DEX reduced fear-potentiated startle to the CS+ only in PTSD individuals.
Michopoulos *et al*., [61]	24 PTSD, 39 controls	0.5 mg dose of DEX, Placebo (counterbalanced)	DEX is an agonist of glucocorticoid receptors	Acquisition, Extinction	Geometric figures/ Air blast	FPS	PTSD and control groups showed significant FPS to the CS+, in the acquisition phase, during placebo and DEX.treatments. Controls showed a significant decrease in FPS from early to late extinction, whereas the PTSD group did not. DEX administration reversed extinction and safety discrimination deficits in the PTSD group.
Inslicht *et al*., [53]	28 PTSD - HC, 28 PTSD - DCS, 28 PTSD - Placebo	25 mg of HC, 50 mg of DCS, Placebo	HC is an agonist of glucocorticoid receptors and annexin A1, a protein involved in the regulation of HPA axis	Acquisition Extinction Recall	Geometric figures/ Electric shock	SCR	A single dose of HC or DCS facilitated fear extinction learning in PTSD individuals.

**Abbreviations:** PTSD = Post-traumatic stress disorder; DEX = dexamethasone; HC = Hydrocortisone; DCS = d-cycloserine; CS-US = conditioned stimulus-unconditioned stimulus; EMG = electromyography; FPS = fear-potentiated startle; SCR = skin conductance response.

## LIST OF ABBREVIATIONS

AD: Alzheimer's Disease
ALS: Amyotrophic Lateral Sclerosis
BDNF: Brain-derived Neurotrophic Factor
CS-: Conditioned Stimulus Never Paired with the US
CS+: Conditioned Stimulus Paired with the US
DCS: D-Cycloserine
DEX: Dexamethasone
fMRI: Functional Magnetic Resonance Imaging
FPS: Fear-potentiated Startle
GRs: Glucocorticoid Receptors
HC: Hydrocortisone
HPA: Hypothalamic-pituitary-adrenal
MEG: Magnetoencephalography
MRs: Mineralocorticoid Receptors
PTSD: Post-traumatic Stress Disorder
SAI: Short-latency Afferent Inhibition
SCR: Skin Conductance Response
TMS: Transcranial Magnetic Stimulation
US: Unconditioned Stimulus
